# On the Death Wound of General Abercrombie

**Published:** 1804-09-01

**Authors:** 


### *To the Editors of the Medical and Physical Journal.*

GENTLEMEN,

A Real Campaigner from Egypt, endeavouring to understand the flighty proem of the Old Campaigner, p. 173, in your last Number, is completely "galled by the miscellany," finds himself wholly incapable of comprehending it. While the fall of the gallant veteran, or what is by far a greater character, of the humane general, has been universally deplored, your Campaigner is perhaps the first who has made it any matter of wonder that the old general should have fallen a victim to a dreadful wound in the arid climate of Egypt.

Mr. Gilham informed Sir Ralph Abercrombie, on examining the wound, that he discovered a ball, and proposed its extraction. On this the General said he sensibly felt it, and cheerfully consented to the operation ; which Mr. Gilham was commencing, when a Field Inspector arriving, and differing in opinion with him, the General was taken to the fleet, where he died, aboard the Admiral's ship.

Mr. Robinson, Inspector of Hospitals, found the ball after death in the situation where Mr. Gilham had declared it, namely, in the *cervix femoris*, near the *trochanter major*.

*Fleet Street, August* 15, 1804.

